# Interpretable artificial intelligence-based app assists inexperienced radiologists in diagnosing biliary atresia from sonographic gallbladder images

**DOI:** 10.1186/s12916-024-03247-9

**Published:** 2024-01-25

**Authors:** Wenying Zhou, Zejun Ye, Guangliang Huang, Xiaoer Zhang, Ming Xu, Baoxian Liu, Bowen Zhuang, Zijian Tang, Shan Wang, Dan Chen, Yunxiang Pan, Xiaoyan Xie, Ruixuan Wang, Luyao Zhou

**Affiliations:** 1 Department of Medical Ultrasonics, Institute for Diagnostic and Interventional Ultrasound, The First Affiliated Hospital, Sun Yat-Sen University, No. 58, Zhongshan Er Road, Guangzhou, 510080 People’s Republic of China; 2 School of Computer Science and Engineering, Sun Yat-Sen University, No. 132, East Outer Ring Road, Guangzhou, 510006 People’s Republic of China; 3 Department of Ultrasound, Shenzhen Children’s Hospital, No. 7019, Yitian Road, Futian District, Shenzhen, 518026 People’s Republic of China; 4 Department of Ultrasound, Guangdong Women and Children’s Hospital, No. 521 Xingnan Avenue, Panyu District, Guangzhou, 511400 People’s Republic of China

**Keywords:** Biliary atresia, Deep learning, Smartphone app, Gallbladder, Ultrasonography

## Abstract

**Background:**

A previously trained deep learning-based smartphone app provides an artificial intelligence solution to help diagnose biliary atresia from sonographic gallbladder images, but it might be impractical to launch it in real clinical settings. This study aimed to redevelop a new model using original sonographic images and their derived smartphone photos and then test the new model’s performance in assisting radiologists with different experiences to detect biliary atresia in real-world mimic settings.

**Methods:**

A new model was first trained retrospectively using 3659 original sonographic gallbladder images and their derived 51,226 smartphone photos and tested on 11,410 external validation smartphone photos. Afterward, the new model was tested in 333 prospectively collected sonographic gallbladder videos from 207 infants by 14 inexperienced radiologists (9 juniors and 5 seniors) and 4 experienced pediatric radiologists in real-world mimic settings. Diagnostic performance was expressed as the area under the receiver operating characteristic curve (AUC).

**Results:**

The new model outperformed the previously published model in diagnosing BA on the external validation set (AUC 0.924 vs 0.908, *P* = 0.004) with higher consistency (kappa value 0.708 vs 0.609). When tested in real-world mimic settings using 333 sonographic gallbladder videos, the new model performed comparable to experienced pediatric radiologists (average AUC 0.860 vs 0.876) and outperformed junior radiologists (average AUC 0.838 vs 0.773) and senior radiologists (average AUC 0.829 vs 0.749). Furthermore, the new model could aid both junior and senior radiologists to improve their diagnostic performances, with the average AUC increasing from 0.773 to 0.835 for junior radiologists and from 0.749 to 0.805 for senior radiologists.

**Conclusions:**

The interpretable app-based model showed robust and satisfactory performance in diagnosing biliary atresia, and it could aid radiologists with limited experiences to improve their diagnostic performances in real-world mimic settings.

**Supplementary Information:**

The online version contains supplementary material available at 10.1186/s12916-024-03247-9.

## Background

Biliary atresia (BA) is one of the most serious infantile cholestatic disease, which can lead to end-stage liver failure or even death without timely diagnosis and surgical treatment [1, 2]. Reliable detection of patients with BA in the early stage is the key to good prognosis [2, 3]. Routine clinical diagnosis of BA among suspected infants depends on ultrasound (US) examination [4–15]. Gallbladder morphology is one of the two most helpful US features (the other is triangular cord thickness) in the detection of BA [1, 4–6, 12, 16, 17], with sensitivity and specificity higher than 90% [12] in experienced hands. However, due to the fact that BA is a rare disease, there is a lack of experts who are good at US diagnosis of BA, and it is a subjective task to accurately identify abnormal gallbladders for many radiologists who are in primary hospitals and lack experience in the diagnosis of BA with US examination.

Recently, an ensemble deep learning model for the artificial intelligence (AI) diagnosis of BA was developed based on US gallbladder images and yielded expert-level performances [18]. However, for the protection of medical data and machines, the vast majority of US machines in hospitals are not connected to the Internet. Consequently, it makes the application of the deep learning model a time-consuming task due to the fact that original US gallbladder images should first be extracted from the US machine system. As a result, it will greatly weaken the radiologists’ willingness to use the model in real clinical settings. In order to simplify the application process, the ensemble deep learning model was further structured into a smartphone app [18], which may potentially provide assistance to radiologists who lack experience in the diagnosis of BA even in underdeveloped regions without robust internet infrastructure.

However, the previously published model was trained not with smartphone photos of gallbladder images but with original US gallbladder images. The image quality of smartphone photos would be inevitably affected by the imaging process, such as noise inclusion or shape and texture deformation [18, 19]. The introduction of test noise during the photo taking process poses several challenges to the stability of the app’s performance, resulting in significant drops in the accuracy and consistency of the app when tested in the real world [20]. New training strategies with more samples are needed to improve the generalization of the smartphone app model. Also, without interpretable outputs, the previous app-based model might not be trusted by radiologists [21].

In addition, it is necessary to validate this smartphone app in real-world mimic settings with various test noise levels or ambient interference presented. In clinical practice, radiologists would rather scan the whole gallbladder dynamically than observe a single frame of gallbladder images in the diagnosis of BA. Even if the AI approach affords high-performance, real­world decisions should be supervised by radiologists for the reasons of safety and accountability. It should be investigated whether an AI­ assisted model can help radiologists with different experiences improve the diagnostic performance of BA in real-world settings.

In this study, we first retrain an interpretable AI-based app model using the original US gallbladder images and their smartphone photos to diagnose BA. Afterwards, we investigated whether radiologists with different experiences could improve their diagnostic performance with the assistance of the interpretable AI-based app in real-world mimic settings.

## Methods

This study was approved by the Research Ethics Committee of the First Affiliated Hospital of Sun Yat­sen University. Written informed parental consent was obtained before prospectively collecting the US gallbladder images and videos from each infant. This study followed the Standards for Reporting of Diagnostic Accuracy (STARD) guidelines for diagnostic studies and the checklist for artificial intelligence in medical imaging by Mongan 2020 [22].

### Study sample for model training and external evaluation

**Fig. 1:**
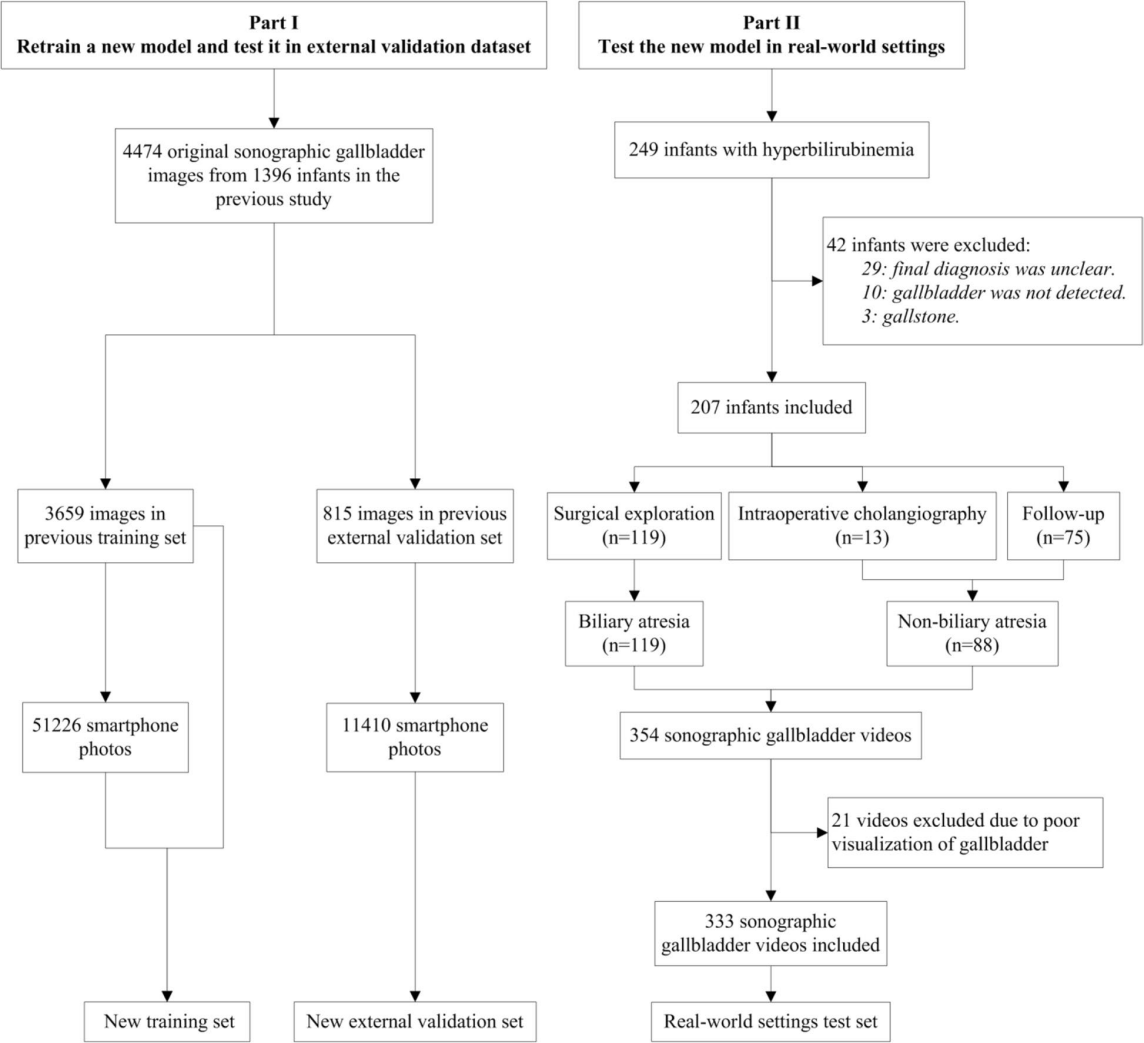
Flow diagram of the inclusion criteria for the study sample

In the first part of this study, a new model was trained with both original US gallbladder images and smartphone photos of US gallbladder images. The initial data set consisted of 4474 original US gallbladder images from 1396 infants derived from a previous study [18], including 3659 images in the training set and 815 images in the external validation set. More details about the dataset were provided in Additional file 1: Method S1. The ground truth label of each image was obtained based on the reference tests for the infant: intraoperative cholangiography, surgical exploration, or jaundice-free after follow-up. Seven radiologists were recruited to take photos of original US gallbladder images with different types of smartphones (Additional file 1: Table S1). The requirements for taking photos with a smartphone are shown in Additional file 1: Method S2. Each radiologist took 2 rounds of photos with a 1-month interval in between, generating a total of 62,636 smartphone photos. A set comprising 3659 original US images and 51,226 corresponding smartphone photos was used to train the new model. The rest of the 11,410 smartphone photos were used to test the new model (Fig. 1, Part I).

### New model equipped in the new smartphone app

**Fig. 2:**
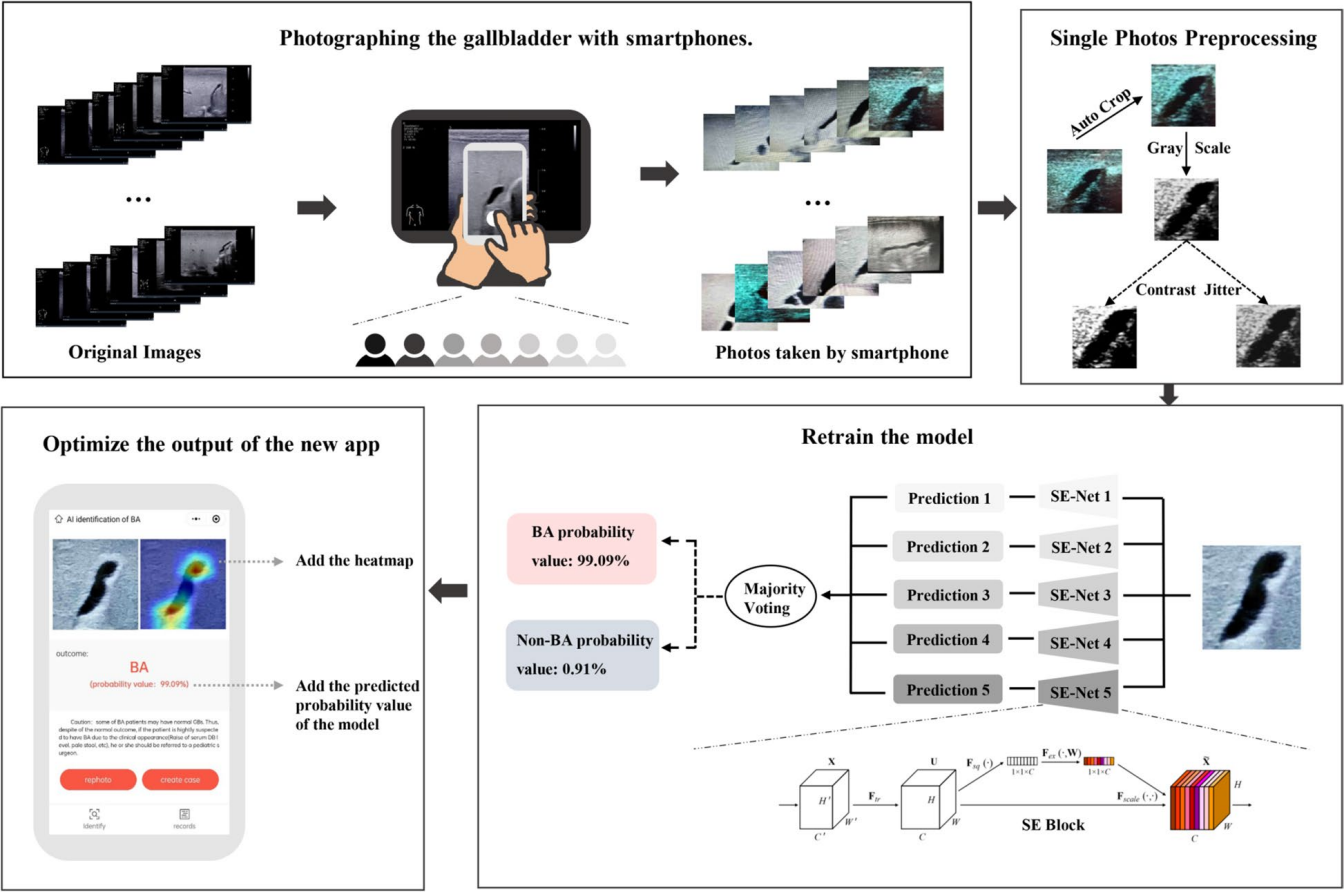
Study profile on the new smartphone app trained with original sonographic gallbladder images and smartphone photos taken by seven radiologists. Seven radiologists used smartphones to take pictures of the original sonographic gallbladder images. Smartphone photos were preprocessed before being inputted into the model for training. Through the fivefold cross-validation method, the new model was trained and finally outputted the predictions and probabilities for the test images. The optimized smartphone app outputted both the heatmap and the prediction probability at the same time

In the first part of this study, a deep convolutional neural network (Se-ResNet-152) [23] was adopted as the architecture of the model. Firstly, we defined a rectangular region of interest covering the gallbladder on the US image to eliminate the interference of irrelevant information from the non-gallbladder areas. Several data augmentation techniques were subsequently implemented on the training dataset, including RandomResizedCrop, RandomHorizontalFlip, and ColorJitter. Then, a fivefold cross-validation method was used to build an ensemble model for the diagnosis of BA (Fig. 2). The ensemble model would output a binary predictive diagnosis (BA or non-BA) for each test image. More details about data pre-processing and model training can be found in Additional file 1: Method S3 and Method S4, respectively.

Previously, only the predicted diagnosis was presented in the output interface of the smartphone app [18]. In order to increase the transparency of the smartphone app, the predicted probability and the heatmap produced by the class activation mapping (CAM) trials [24] of the model were presented along with the predicted diagnosis in this study (Fig. 2).

### Patients in real-world mimic settings

In the second part of this study, the new smartphone app model was tested in real-world mimic settings. Between July 2019 and August 2021, a total of 182 infants with jaundice were prospectively enrolled at the First Affiliated Hospital of Sun Yat­sen University. The inclusion criteria for infants were as follows: (1) infants were younger than 1 year old with conjugated hyperbilirubinemia, which was defined as the serum direct bilirubin level > 17.1 μmol/L and the ratio of direct to total bilirubin level > 20% [25]; (2) BA could not be ruled out based on their symptoms and signs; and (3) US gallbladder images of infants were never used for model training. Infants who were unable to cooperate with the US examination or whose gallbladder was not detected by the high-frequency US transducer were excluded. All included infants had definite final diagnoses of BA or non-BA, which were confirmed by surgical exploration, intraoperative cholangiography, or follow-up. Sixty-seven infants from other 6 hospitals were also prospectively enrolled from January 2020 to January 2023 for the robustness evaluation of the new model. The 6 participating hospitals can be found in Additional file 1: Method S5. Forty-two patients were excluded for the following reasons: (1) final diagnosis was unclear (*n* = 29), (2) gallbladder could not be detected by US (*n* = 10), and (3) infants with gallstone (*n* = 3). Finally, 207 infants were included in this study (Fig. 1, Part II). More details about the sample size calculation are provided in Additional file 1: Method S6.

### Sonographic gallbladder video acquirement

Radiologists (> 10 years of experience with abdominal US) from different hospitals recorded all gallbladder videos. Infants were not fed for at least 2 h before the US examination and were kept quiet by feeding during the examination. The scan procedure was the same as those previously reported [6, 26], with the additional step to store the dynamic gallbladder videos. More details about the acquisition requirements for US gallbladder videos can be seen in Additional file 1: Method S7. Two examples of qualified US gallbladder videos were also presented in the supplementary material (Additional file 2: Video S1 and Additional file 3: Video S2). Two different linear array transducers (SL10-2 and SL15-4) from the same US equipment (SuperSonic Imagine, Aix-en-Provence, France) were used to generate the US images in the First Affiliated Hospital of Sun Yat­sen University. For each infant, only one video generated by each transducer was reserved for analysis. Therefore, there would be 2 gallbladder videos obtained from two different linear array transducers for each infant from this center. Infants from the other six hospitals were examined with the following US scanners: LOGIQ E8, E9, and E20 (GE); Resona 7OB (Mindray); EPIQ 5 and EPIQ7 (Philips); and Aixplorer (SuperSonic Imagine). All gallbladder videos were obtained with high-frequency transducers (> 10 MHz).

Twenty-one videos with poor imaging quality (severe motion artifacts or refraction artifacts) were excluded after screening by a junior researcher (W.Y.Z., with 3 years of experience with pediatric abdominal US). Finally, 333 gallbladder videos from 207 infants were obtained as the test data.

### Testing in real-world mimic settings

**Fig. 3:**
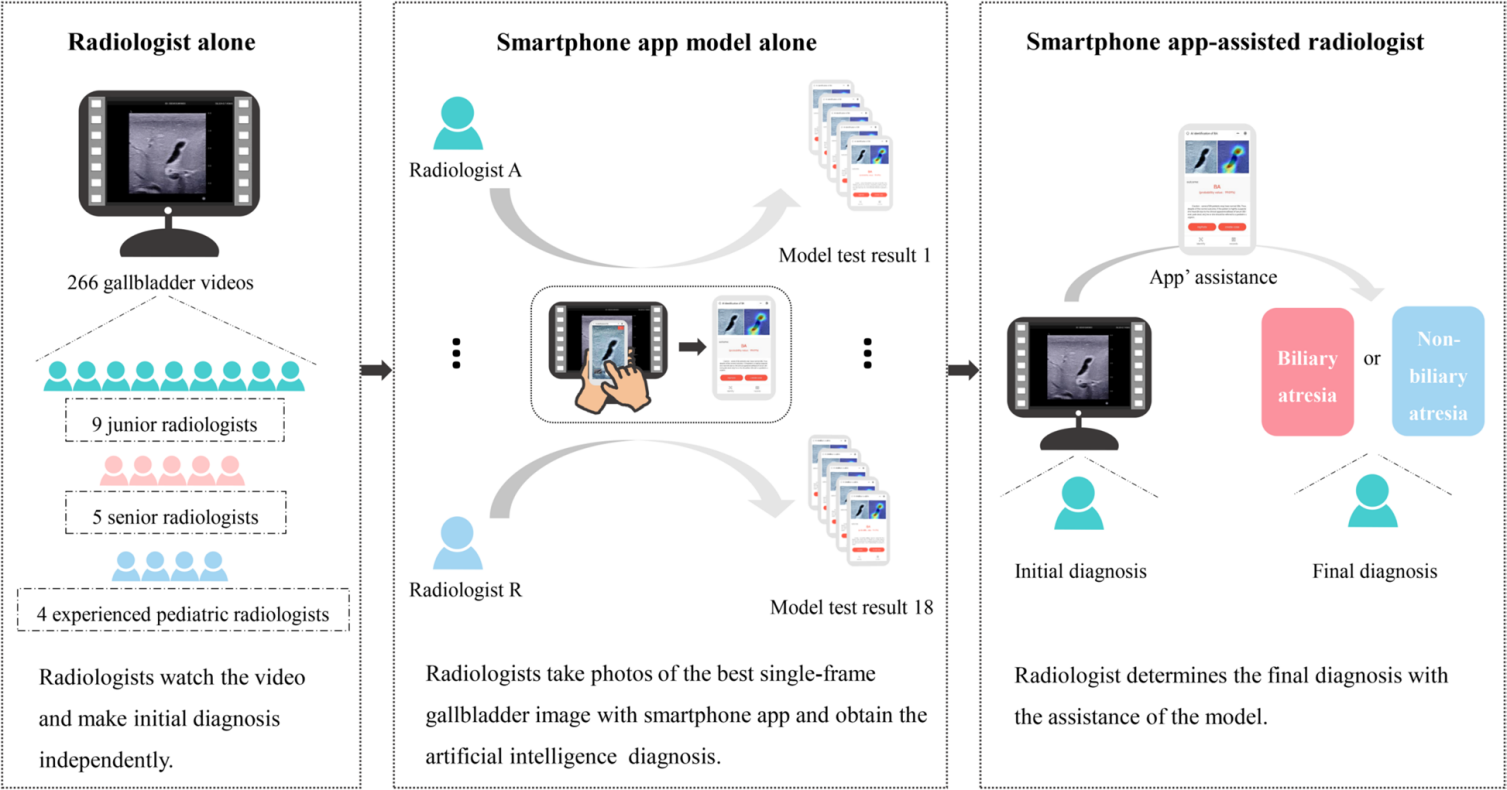
The diagnostic process for radiologists alone, smartphone app alone, and radiologists with smartphone app’s assistance in diagnosing biliary atresia based on 333 prospectively collected sonographic gallbladder videos. Eighteen radiologists with different experiences made the initial diagnosis by watching the sonographic gallbladder video independently. Then, radiologists used the smartphone app to take pictures of the gallbladder images and obtained the diagnosis of the model. Through the combination of their own knowledge and the diagnosis provided by the smartphone app, radiologists made the final diagnosis

A total of 18 radiologists, including 9 junior radiologists (1–2 years of experience in abdominal US), 5 senior radiologists (> 10 years of experience in abdominal US), and 4 experienced pediatric radiologists (> 5 years of experience in pediatric US), were recruited to review the gallbladder videos and make diagnoses independently (Fig. 3). Among them, 9 junior radiologists and 5 senior radiologists were recruited from the First Affiliated Hospital of Sun Yat­sen University, and 4 experienced pediatric radiologists were recruited from Guangdong Women and Children’s Hospital and Shenzhen Children’s Hospital. Except for the 4 experienced pediatric radiologists, other radiologists lacked experience in diagnosing BA with real-time US before participating in this study. Before starting to read any images or videos, those inexperienced radiologists would be briefed on the features of normal (indicated non-BA) and abnormal (indicated BA) US gallbladders with numbers of typical image and video examples displayed simultaneously. An abnormal gallbladder was defined as one of the following [5, 6, 11, 27, 28]: filled gallbladder lumen less than 1.5 cm in length; the gallbladder lacks a smooth echogenic mucosal lining and has indistinct walls and irregular/lobular contour (Additional file 1: Fig. S1). Each video without any patient identity information was presented in a random order. All these 18 radiologists have not read any of the patient’s images or videos before attending this study and were blinded to any other patient’s information during their diagnoses. The laptops they used to play the videos and the smartphones they used to take photos are listed in Additional file 1: Table S2.

In this part, a three-step diagnostic process was conducted to evaluate the performance of the new model in assisting radiologists in diagnosing BA (Fig. 3). Firstly, each radiologist reviewed the dynamic gallbladder video alone and obtained the initial independent diagnosis (hereafter, initial diagnosis). In the process of independent diagnosis, radiologists could watch the videos repeatedly. Secondly, each radiologist selected the best single-frame gallbladder image from the video, took a picture with the smartphone app, sent it to the model for AI diagnosis, and recorded the reference diagnosis given by the app (hereafter, AI diagnosis). Finally, based on the reference diagnosis provided by the smartphone app, the radiologists chose to adhere to their initial diagnosis or adopt the diagnosis from the smartphone app as the final diagnosis (hereafter, final diagnosis). The initial diagnosis, AI diagnosis, and final assisted diagnosis were recorded.

In order to explore the changes in radiologists’ diagnostic confidence with or without the aid of the smartphone app, 4-scale self-confidence should be given by each radiologist for the initial and final diagnosis, with “1” to “4,” representing “definitely non-BA,” “probably non-BA,” “probably BA,” and “definitely BA,” respectively.

### Statistical analysis

The continuous variables were first tested for normality using a Kolmogorov–Smirnov test. Differences between the BA group and the non-BA group were compared using the *t* test for normally distributed variables, the Wilcoxon rank test for skewed variables, and the chi-squared test for categorical variables. The diagnostic performance was expressed as the area under the receiver operating characteristic curve (AUC). Differences between various AUCs were compared using a Delong test (single comparison) or paired *T*-test (multiple comparisons). Fleiss’ multirater kappa was used to assess the agreement of binary classification results between multiple diagnoses. The agreement was graded as follows: poor (*κ* < 0.20), moderate (*κ* = 0.20 to < 0.40), fair (*κ* = 0.40 to < 0.60), good (*κ* = 0.60 to < 0.80), or very good (*κ* = 0.80–1.00). The AUC of the radiologists alone was used for two comparisons (radiologist alone vs model, radiologist alone vs AI-assisted radiologist). Therefore, a Bonferroni correction was performed to lower the significance threshold to 0.025. For the other comparisons, *P* < 0.05 was considered a statistically significant difference. All statistical tests were two-sided. The analyses were performed with the MedCalc Statistical Software version 15.2.2 (MedCalc) and SPSS software package version 25 (IBM).

## Results

### External validation of the new model

In the first part of this study, the new model yielded an average sensitivity (recall) of 83.3% [95% confidence interval (CI) 82.0–84.7%] and an average precision of 69.2% (95% CI 65.5–72.8%), while the previous published model yielded an average sensitivity (recall) of 70.4% (95% CI 64.7–76.1%) and an average precision of 74.7% (95% CI 72.4–77.0%) (Additional file 1: Fig. S2). The diagnostic performance of the new model [average AUC 0.924 (95% CI 0.918–0.930)] was better than that of the previous published model [average AUC 0.908 (95% CI 0.896–0.920)] (*P* = 0.004) in diagnosing BA on the new external test set. When tested on seven batches of smartphone photos taken by seven radiologists in the first round, the kappa value of the new smartphone app model was 0.708, the agreement of which was more satisfactory than that of the previous one (kappa value 0.609).

### Clinical characteristics of patients in real-world mimic settings test

There were 119 infants with BA and 88 infants without BA in the clinical test cohort. Among the 88 infants without BA, 2 were diagnosed with congenital biliary dilatation, 2 were diagnosed as neonatal intrahepatic cholestasis caused by citrin deficiency, 1 was diagnosed with neonatal hemolysis, and the remaining 83 were diagnosed with neonatal hepatitis syndrome. Of the 333 videos included prospectively in the second part, 189 videos belonged to 119 infants with BA and 144 videos belonged to 88 infants with non-BA. No significant differences were observed between the BA group and the non-BA group in terms of sex, age, and total bilirubin levels (all *P* > 0.05), but there were differences in terms of direct bilirubin levels (*P* < 0.001), alanine aminotransferase levels (*P* = 0.002), aspartate aminotransferase levels (*P* = 0.002), and γ-glutamyltransferase levels (*P* < 0.001) (Table 1).

**Table 1:** Patient characteristics at the time of ultrasound examination

Characteristics	BA group (*n* = 119)	Non-BA group (*n* = 88)	*P* value
Male-to-female ratio^a^	60:59	53:35	0.16
Age (days)^b^	53 (40, 68)	50 (33, 76)	0.73
TB (mmol/L)^b^	151.5 (125.0, 194.9)	142.1 (99.2, 204.4)	0.37
DB (mmol/L)^b^	98.1 (75.8, 128.2)	66.0 (25.2, 103.6)	< 0.001
ALT (U/L)^b^	133.0 (70.0, 249.0)	73.0 (26.0, 177.0)	0.002
AST (U/L)^b^	201.0 (128.0, 331.0)	101.0 (47.0, 240.0)	0.002
GGT (U/L)^b^	377.0 (269.0, 687.0)	144.0 (80.3, 231.0)	< 0.001

*BA* Biliary atresia, *TB* Total bilirubin, *DB* Direct bilirubin, *ALT* Indicates alanine aminotransferase, *AST* Aspartate aminotransferase, *GGT* γ-glutamyl transferase

^a^Data are numbers of patients. The chi-square test was used to test the sex distribution between the two groups

^b^Data are medians with interquartile ranges reported in parentheses. The Mann–Whitney *U* test was used to compare the variables between the two groups

### Testing in real-world mimic settings

The average AUCs of radiologists’ initial diagnosis were 0.773 (95% CI 0.734–0.812) for 9 junior radiologists (Table 2), 0.749 (95% CI 0.669–0.828) for 5 senior radiologists (Table 3), and 0.876 (95% CI 0.830–0.922) for 4 experienced pediatric radiologists (Table 4). When tested with smartphone photos of US gallbladder images taken by the above radiologists, the average AUC of the new smartphone app model was 0.838 (95% CI 0.827–0.850) by junior radiologists (Table 2), 0.829 (95% CI 0.784–0.875) by senior radiologists (Table 3), and 0.860 (95% CI 0.7950.924) by experienced pediatric radiologists (Table 4). The AUC achieved by the new model was comparable to that of 3 of 4 experienced pediatric radiologists (*P* < 0.025 for 1 of 4 experienced pediatric radiologists, Table 4) and significantly better than that of most inexperienced radiologists alone (*P* < 0.025 for 10 of the 14 radiologists) (Tables 2 and 3). Furthermore, the kappa value of this smartphone app model was 0.707 (good) across 18 tests.

**Table 2:** The diagnostic performance of junior radiologists alone, smartphone app alone, and junior radiologists with smartphone app’s assistance in diagnosing biliary atresia

Radiologist^a^	AUC	Sensitivity (%)	Specificity (%)	Accuracy (%)	*P*1 value^#^	*P*2 value^#^	Not gallbladder
A-initial	0.798 (0.751, 0.840)	78.3 (71.7, 84.0)	81.3 (73.9, 87.3)	79.6 (70.3, 89.8)	0.11	0.01	3
Model	0.838 (0.794, 0.876)	87.8 (82.3, 92.1)	79.9 (72.4, 86.1)	84.4 (74.8, 94.9)	–	–	–
A-final	0.863 (0.821, 0.898)	90.0 (84.7, 93.8)	82.6 (75.4, 88.4)	86.8 (77.1, 97.4)	–	–	–
B-initial	0.784 (0.735, 0.827)	85.2 (79.3, 89.9)	71.5 (63.4, 78.7)	79.3 (70.0, 89.4)	0.02	0.09	9
Model	0.841 (0.797, 0.879)	88.4 (82.9, 92.6)	79.9 (72.4, 86.1)	84.7 (75.1, 95.2)	–	–	–
B-final	0.819 (0.773, 0.859)	89.4 (84.1, 93.4)	74.3 (66.4, 81.2)	82.9 (73.4, 93.3)	–	–	–
C-initial	0.853 (0.811, 0.890)	89.4 (84.1, 93.4)	81.3 (73.9, 87.3)	85.9 (76.2, 96.4)	0.66	0.87	5
Model	0.850 (0.807, 0.887)	91.5 (86.6, 95.1)	78.5 (70.9, 84.9)	85.9 (76.2, 96.4)	–	–	–
C-final	0.860 (0.818, 0.896)	86.4 (81.0, 90.8)	87.4 (80.3, 92.6)	86.5 (76.8, 97.1)	–	–	–
D-initial	0.725 (0.674, 0.772)	80.4 (74.0, 85.8)	64.6 (56.2, 72.4)	73.6 (64.7, 83.4)	< 0.001	< 0.001	7
Model	0.849 (0.806, 0.886)	92.1 (87.2, 95.5)	77. 8 (70.1, 84.3)	85.9 (76.2, 96.4)	–	–	–
D-final	0.810 (0.764, 0.851)	88.4 (82.9, 92.6)	73.6 (65.6, 80.6)	82.0 (72.5, 92.3)	–	–	–
E-initial	0.777 (0.728, 0.821)	89.4 (84.1, 93.4)	66.0 (57.6, 73.7)	79.3 (70.0, 89.4)	0.12	0.06	8
Model	0.820 (0.775, 0.860)	88.4 (82.9, 92.6)	75.7 (67.9, 82.4)	82.9 (73.4, 93.3)	–	–	–
E-final	0.828 (0.783, 0.867)	90.0 (84.7, 93.8)	75.7 (67.9, 82.4)	83.8 (74.2, 94.2)	–	–	–
F-initial	0.821 (0.776, 0.861)	92.1 (87.2, 95.5)	72.2 (64.2, 79.4)	83.5 (74.0, 93.9)	0.02	0.003	8
Model	0.866 (0.824, 0.900)	90.5 (85.4, 94.3)	82.6 (75.4, 88.4)	87.1 (77.4, 97.7)	–	–	–
F-final	0.876 (0.836, 0.909)	90.5 (85.4, 94.3)	84.7 (77.8, 90.2)	88.0 (78.2, 98.7)	–	–	–
G-initial	0.769 (0.720, 0.813)	85.7 (79.9, 90.4)	68.1 (59.8, 75.6)	78.1 (68.9, 88.2)	0.01	0.01	7
Model	0.835 (0.791, 0.873)	87.8 (82.3, 92.1)	79.2 (71.6, 85.5)	84.1 (74.5, 94.5)	–	–	–
G-final	0.806 (0.760, 0.847)	88.4 (82.9, 92.6)	72.9 (64.9, 80.0)	81.7 (72.3, 92.0)	–	–	–
H-initial	0.743 (0.693, 0.789)	77. 8 (71.2, 83.5)	70.8 (62.7, 78.1)	74.8 (65.8, 84.7)	0.01	< 0.001	13
Model	0.817 (0.771, 0.857)	86.2 (80.5, 90.8)	77.1 (69.3, 83.7)	82.3 (72.8, 92.7)	–	–	–
H-final	0.820 (0.775, 0.860)	86.2 (80.5, 90.8)	77. 8 (70.1, 84.3)	82.6 (73.1, 92.9)	–	–	–
I-initial	0.686 (0.633, 0.735)	63.5 (56.2, 70.4)	73.6 (65.6, 80.6)	67.9 (59.3, 77.3)	< 0.001	< 0.001	21
Model	0.830 (0.785, 0.868)	86.8 (81.1, 91.3)	79.2 (71.6, 85.5)	83.5 (74.0, 93.9)	–	–	–
I-final	0.829 (0.784, 0.868)	87.3 (81.7, 91.7)	78.5 (70.9, 84.9)	83.5 (74.0, 93.9)	–	–	–

95% confidence intervals are included in brackets

^a^Nine junior radiologists were labeled “A” to “I”. “-initial” represents the diagnosis of the radiologist alone; “Model” represents the diagnosis of the smartphone app tested with the photos taken by the relevant radiologist; “-final” represents the diagnosis of the radiologist with smartphone app’s assistance

^#^The* P*1 values were from the comparison between the AUC of the radiologists alone and the AUCs of the smartphone app alone. The *P*2 values were from the comparison between the AUC of the radiologists alone and the AUCs of smartphone app-assisted radiologists. Differences between various AUCs were compared using a Delong test

**Table 3:** The diagnostic performance of senior radiologists alone, smartphone app alone, and senior radiologists with smartphone app’s assistance in diagnosing biliary atresia

Radiologist^a^	AUC	Sensitivity (%)	Specificity (%)	Accuracy (%)	*P*1 value^#^	*P*2 value^#^	Not gallbladder
J-initial	0.816 (0.770, 0.856)	88.9 (83.5, 93.0)	74.3 (66.4, 81.2)	82.6 (73.1, 92.9)	0.01	0.18	0
Model	0.874 (0.834, 0.908)	91.5 (86.6, 95.1)	83.3 (76.2, 89.0)	88.0 (78.2, 98.7)	–	–	–
J-final	0.841 (0.797, 0.879)	88.4 (82.9, 92.6)	79.9 (72.4, 86.1)	84.7 (78.1, 95.2)	–	–	–
K-initial	0.714 (0.662, 0.762)	94.2 (89.8, 97.1)	48.6 (40.2, 57.1)	66.1 (57.6, 75.4)	< 0.001	0.01	2
Model	0.859 (0.817, 0.895)	90.0 (84.7, 93.8)	81.9 (74.7, 87.9)	79.3 (70.0, 89.4)	–	–	–
K-final	0.753 (0.703, 0.798)	93.7 (89.2, 96.7)	56.9 (48.4, 65.2)	81.4 (72.0, 91.7)	–	–	–
L-initial	0.670 (0.616, 0.720)	60.3 (53.0, 67.3)	73.6 (65.6, 80.6)	74.5 (65.5, 84.3)	< 0.001	< 0.001	8
Model	0.785 (0.737, 0.828)	84.1 (78.1, 89.0)	72.9 (64.9, 80.0)	86.5 (76.8, 97.1)	–	–	–
L-final	0.810 (0.763, 0.850)	84.1 (78.1, 89.0)	77.8 (70.1, 84.3)	77.8 (68.6, 87.9)	–	–	–
M-initial	0.731 (0.680, 0.778)	58.7 (51.4, 65.8)	87.5 (81.0, 92.4)	71.2 (62.4, 80.8)	0.001	0.002	4
Model	0.818 (0.773, 0.858)	87.3 (81.7, 91.7)	76.4 (68.6, 83.1)	82.6 (73.1, 92.9)	–	–	–
M-final	0.791 (0.744, 0.834)	71.4 (64.4, 77.8)	86.8 (80.2, 91.9)	78.1 (68.9, 88.2)	–	–	–
N-initial	0.812 (0.766, 0.853)	88.9 (83.5, 93.0)	73.6 (65.6, 80.6)	82.3 (72.8, 92.7)	0.91	0.08	3
Model	0.810 (0.764, 0.851)	90.5 (85.4, 94.3)	71.5 (63.4, 78.7)	82.3 (72.8, 92.7)	–		–
N-final	0.830 (0.785, 0.869)	91.0 (86.0, 94.7)	75.0 (67.1, 81.8)	84.1 (74.5, 94.5)	–		–

95% confidence intervals are included in brackets

^a^Five senior radiologists were labeled “J” to “N”. “-initial” represents the diagnosis of the radiologist alone; “Model” represents the diagnosis of the smartphone app tested with the photos taken by the relevant radiologist; “-final” represents the diagnosis of the radiologist with smartphone app’s assistance

^#^The *P*1 values were from the comparison between the AUC of the radiologists alone and the AUCs of the smartphone app alone. The *P*2 values were from the comparison between the AUC of the radiologists alone and the AUCs of smartphone app-assisted radiologists. Differences between various AUCs were compared using a Delong test

**Table 4:** The diagnostic performance of experienced pediatric radiologists alone, smartphone app alone, and experienced pediatric radiologists with smartphone app’s assistance in diagnosing biliary atresia

Radiologist^a^	AUC	Sensitivity (%)	Specificity (%)	Accuracy (%)	*P*1 value^#^	*P*2 value^#^	Not gallbladder
O-initial	0.903 (0.866, 0.932)	88.9 (83.5, 93.0)	91.7 (85.9, 95.6)	82.9 (73.4, 93.3)	< 0.001	0.16	0
Model	0.831 (0.787, 0.870)	94.7 (90.5, 97.4)	71.5 (63.4, 78.7)	84.7 (75.1, 95.2)	–	–	–
O-final	0.897 (0.859, 0.927)	88.4 (82.9, 92.6)	91.0 (85.1, 95.1)	89.5 (79.6, 100)	–	–	–
P-initial	0.887 (0.848, 0.919)	85.7 (79.9, 90.4)	91.7 (85.9, 95.6)	64.0 (55.7, 73.2)	0.25	0.91	0
Model	0.911 (0.875, 0.939)	94.7 (90.5, 97.4)	87.5 (81.0, 92.4)	91.6 (81.6, 100)	–	–	–
P-final	0.886 (0.847, 0.918)	86.2 (80.5, 90.8)	91.0 (85.1, 95.1)	65.8 (57.3, 75.1)	–	–	–
Q-initial	0.835 (0.791, 0.874)	94.2 (89.8, 97.1)	72.9 (64.9, 80.0)	85.0 (75.4, 95.5)	0.37	0.07	0
Model	0.823 (0.778, 0.863)	93.1 (88.5, 96.3)	71.5 (63.4, 78.7)	83.5 (74.0, 93.9)	–	–	–
Q-final	0.814 (0.768, 0.855)	92.1 (87.2, 95.5)	70.8 (62.7, 78.1)	82.9 (73.4, 93.3)	–	–	–
R-initial	0.878 (0.838, 0.911)	82.5 (76.4, 87.7)	93.1 (87.6, 96.6)	87.1 (77.4, 97.7)	0.77	0.51	0
Model	0.873 (0.832, 0.907)	85.7 (79.9, 90.4)	88.9 (82.6, 93.5)	87.1 (77.4, 97.7)	–	–	–
R-final	0.882 (0.843, 0.915)	84.1 (78.1, 89.0)	92.4 (86.7, 96.1)	87.7 (77.9, 98.3)	–	–	–

95% confidence intervals are included in brackets

^a^Four experienced pediatric radiologists were labeled “O” to “R”. “-initial” represents the diagnosis of the radiologist alone; “Model” represents the diagnosis of the smartphone app tested with the photos taken by the relevant radiologist; “-final” represents the diagnosis of the radiologist with smartphone app’s assistance

^#^The *P*1 values were from the comparison between the AUC of the radiologists alone and the AUCs of the smartphone app alone. The *P*2 values were from the comparison between the AUC of the radiologists alone and the AUCs of smartphone app-assisted radiologists. Differences between various AUCs were compared using a Delong test

**Fig. 4:**
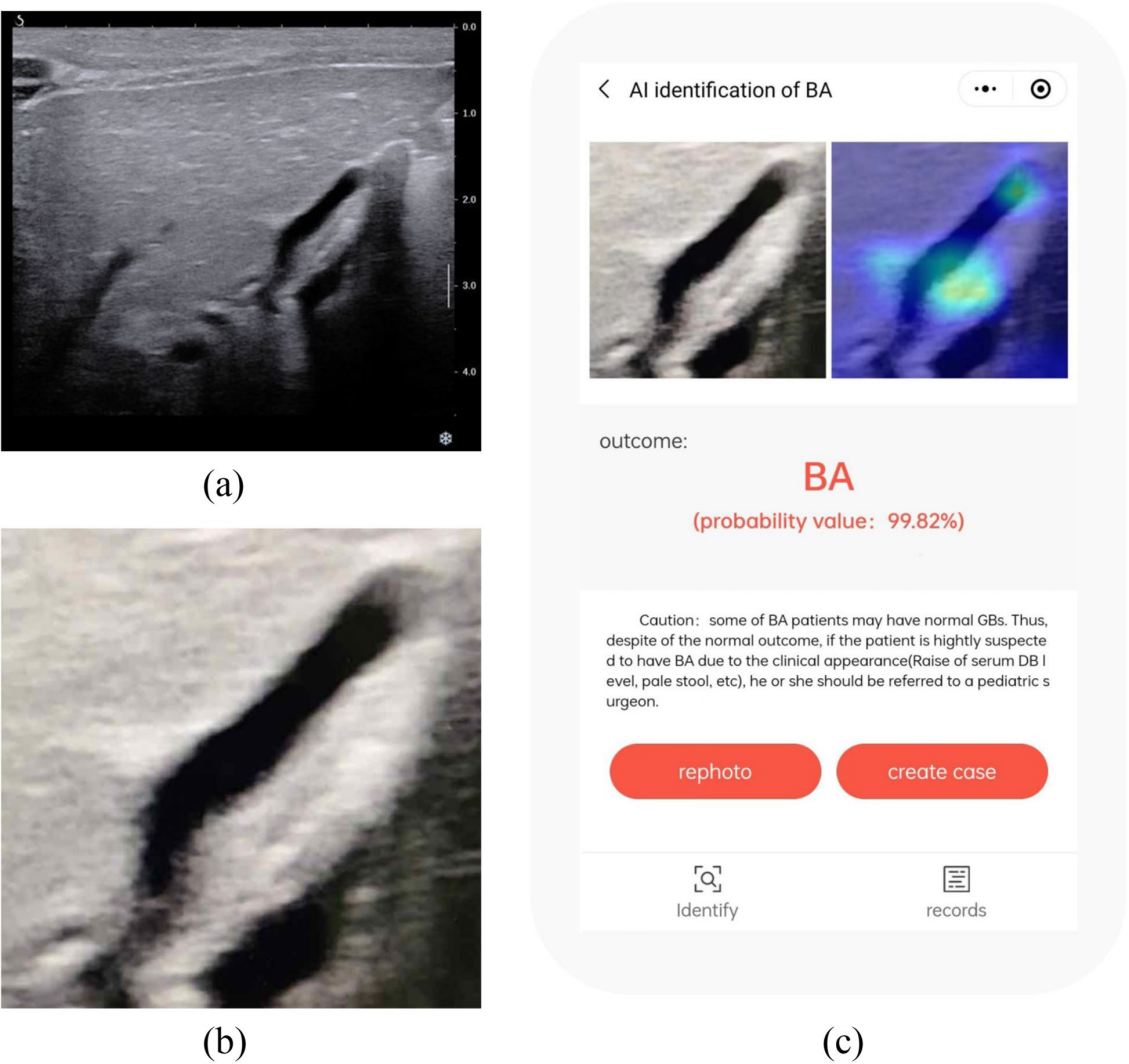
Example of a case where radiologists obtained the correct diagnosis with the assistance of the new smartphone app. **a** An original sonographic gallbladder image selected from the video of a 65-day-old female infant with biliary atresia. Seven radiologists considered the gallbladder to be normal. However, the diagnosis provided by the model obtained from the photos taken by six of these seven radiologists indicated biliary atresia, and the heatmaps all focused on the gallbladder. Finally, five radiologists revised the diagnosis of this infant to biliary atresia. **b** The smartphone photo taken by radiologist J. **c** The output interface of the test result in the new smartphone app for smartphone photo shown in **b**, displaying a probability value of 99.82%. With the assistance of the new smartphone app, radiologist J finally revised the diagnosis of this infant to biliary atresia

With the app’s assistance, the average AUC of 9 junior radiologists was improved from 0.773 to 0.835. The improvement in AUCs for junior radiologists ranged from 0.007 to 0.143 (median 0.055). The average AUC of 5 senior radiologists was improved from 0.749 to 0.805. The improvement in AUCs for senior radiologists ranged from 0.014 to 0.140 (median 0.039). The AUCs of these 14 inexperienced radiologists all improved with the app’s assistance, and 9 of them were statistically different (Tables 2 and 3, Fig. 4, and Additional file 1: Fig. S3). Of note, 7 junior radiologists (Table 2, radiologist A, B, D, E, and G–I) and 2 senior radiologists (Table 3, radiologist L and N) had improved diagnostic sensitivity and specificity with smartphone app assistance.

Only one experienced pediatric radiologist had an increase in AUC with the help of the model (radiologist R, AUC improved from 0.878 to 0.882), while the other three experienced pediatric radiologists’ AUCs decreased slightly, although no statistical difference was observed (Table 4, all *P* > 0.025).

**Fig. 5:**
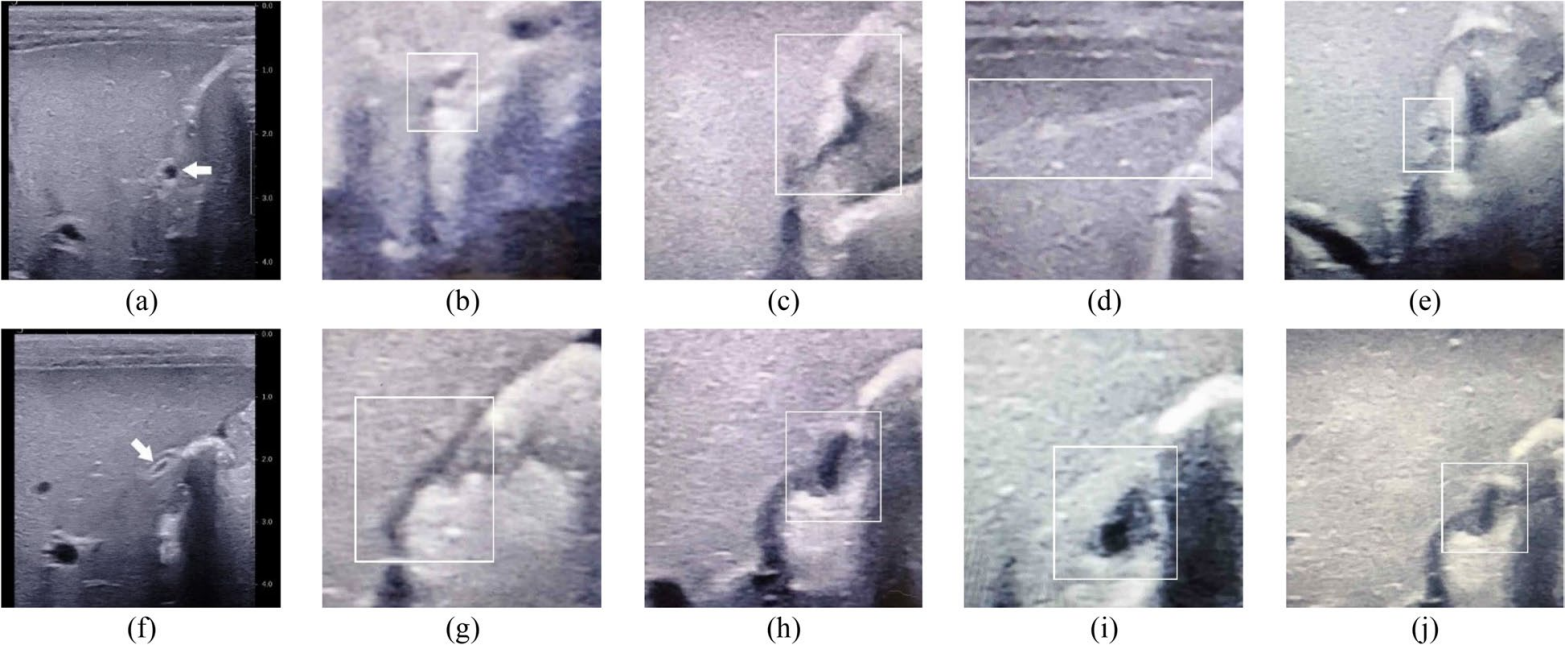
Examples of cases where radiologists consider non-gallbladder as gallbladder. **a**–**e** These belong to a 59-day-old female infant with biliary atresia, in which the arrow in **a** refers to the correct gallbladder (small gallbladder) shown in the original sonographic video. The hepato-intestinal space in **b** and **c**, the blood vessels in **d**, and the gut in **e** are non-gallbladder structures (rectangles) in smartphone photos, which were considered as gallbladder by four radiologists. **f**–**j** These belong to a 58-day-old male infant with non-biliary atresia, in which the arrow in **f** refers to the correct gallbladder (unfilled gallbladder) in the original sonographic video. The hepato-intestinal space in **g** and the gut in **h**–**j** are the non-gallbladder structures (rectangles) in smartphone photos, which were considered as gallbladder by four radiologists. Sonographic gallbladder videos of these two infants are available in Additional file 4: Video S3 for the first infant and in Additional file 5: Video S4 for the second infant

In particular, there were 8.4% videos (28/333) in which the non-gallbladder was mistakenly regarded as gallbladder by some radiologists (Fig. 5), of which 13 belonged to BA and 15 belonged to non-BA. Those non-gallbladder structures contained the hepato-intestinal space, blood vessels, or gut. In these cases, the model was more inclined to diagnose BA (68.0%, 66/97 times). Junior radiologists (81 times/9 radiologists) were more likely to regard non-gallbladder structures as gallbladder than senior radiologists (16 times/5 radiologists) (*P* < 0.001).

### Changes in diagnosis confidence with the assistance of the smartphone app

**Fig. 6:**
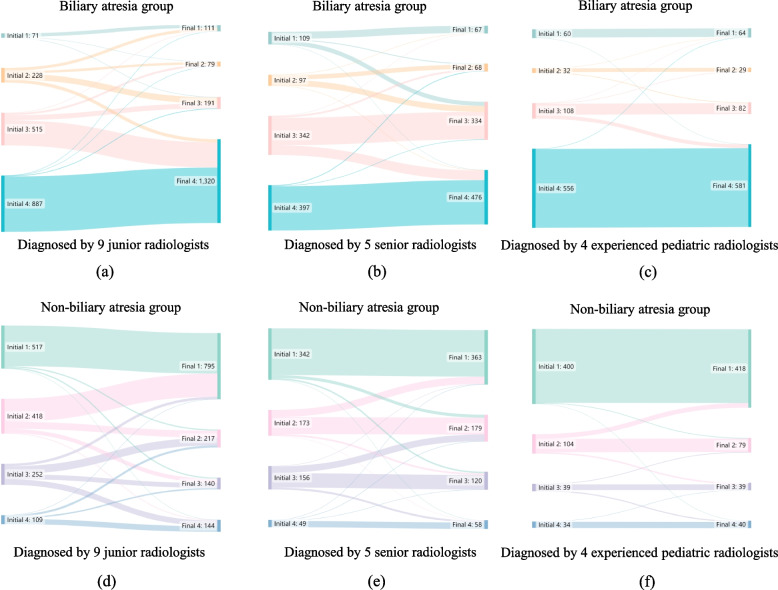
Changes in diagnostic confidence between initial diagnosis made by radiologists alone and final diagnosis made by radiologists with smartphone app’s assistance. **a** Changes in junior radiologists in the cohort with true label biliary atresia. **b** Changes in senior radiologists in the cohort with true label biliary atresia. **c** Changes in experienced pediatric radiologists in the cohort with true label biliary atresia. **d** Changes in junior radiologists in the cohort with true label non-biliary atresia. **e** Changes in senior radiologists in the cohort with true label non-biliary atresia. **f** Changes in experienced pediatric radiologists in the cohort with true label non-biliary atresia. * “Initial” represents independent diagnosis by radiologists, and “Final” represents the radiologist’s diagnosis with the assistance of the model. “1” to “4” represent “definitely non-BA,” “probably non-BA,” “probably BA,” and “definitely BA,” respectively

The change in diagnostic confidence for each radiologist was presented separately in Additional file 1: Table S3, while the change in confidence in the number of diagnoses for radiologists with different experiences is summarized in Fig. 6.

The percentages of cases with changes in diagnostic confidence before and after the app’s assistance were 42.1% (1261/2997) for junior radiologists, 22.6% (377/1665) for senior radiologists, and 6.9% (92/1332) for experienced pediatric radiologists. With the assistance of the app, the diagnostic confidence of most radiologists had been improved in the final diagnosis, especially junior radiologists (Additional file 1: Table S3). In the BA cohort, the percentages of infants with a change from wrong initial diagnosis “1, 2” to correct final diagnosis “3, 4” were 51.5% (154/299) of junior radiologists, 45.9% (94/206) of senior radiologists, and 6.5% (6/92) of experienced pediatric radiologists. On the other hand, the model could also help radiologists correctly identify some non-BA infants who were initially misdiagnosed as BA (from “3, 4” to “1, 2”), and the percentages of infants with this change were 41.8% (151/361) of junior radiologists, 23.4% (48/205) of senior radiologists, and 5.5% (4/73) of experienced pediatric radiologists.

Notably, many patients were correctly converted from “probably” to “definitely” by junior radiologists, senior radiologists, and even experienced pediatric radiologists, of whom their initial diagnosis was correct, as “3” was mostly converted to “4” in the BA cohort, while “2” was mostly converted to “1” in the non-BA cohort (Fig. 6).

However, it must be acknowledged that this model may also mislead the radiologists in missing the diagnosis of BA. In the BA group, the percentage of cases with conversion from “3, 4” to “1, 2” was 3.2% (45/1402) of junior radiologists, 3.1% (23/739) of senior radiologists, and 1.1% (7/664) of experienced pediatric radiologists.

## Discussion

In this study, we redeveloped a deep learning-based smartphone app model with new strategies and expanded samples. The results showed the new model outperformed the previous published one (AUC 0.924 vs 0.908, *P* = 0.004) with higher consistency (kappa value of the new model 0.708 vs the old model 0.609). In addition, the diagnostic performance of the new model was comparable to that of experienced pediatric radiologists (average AUC 0.860 vs 0.876) and significantly superior than that of junior and senior radiologists with limited experience (average AUC 0.838 vs 0.773, and 0.829 vs 0.749, respectively) in real-world mimic settings. More importantly, the new app could aid both junior and senior radiologists with limited experience to improve their diagnostic performance (average AUC increasing from 0.773 to 0.835 for junior radiologist and from 0.749 to 0.805 for senior radiologists, respectively) with increased self-confidence. All these findings showed that this newly developed model has great potential to help inexperienced radiologists diagnose BA in real clinical practice. Therefore, with the application of the new model, there might be a reduction in delayed diagnosis of BA for suspected infants in primary hospitals without experienced radiologists.

Smartphone photos exhibit variability in factors such as camera resolution of the smartphone, viewing angle of photos, and moiré pattern of the screen device during the artificial collection process, making diagnosis substantially more challenging [19]. In this study, we overcome this challenge by collecting a large number of smartphone photos of US gallbladder images to jointly train the model, thereby making the model robust enough to photographic variability. When tested in real-world mimic settings, the new model still performed well among smartphone photos taken by different radiologists. Furthermore, the agreement of the model was graded as good when tested with smartphone photos taken by 18 radiologists, showing the stability of the model in subtle changeable environments among different operators. All these findings reveal the generalization and robustness of this new smartphone app in real-world mimic settings.

In this study, we also optimize the model by adding prediction probability and heatmap to the output interface. Radiologists would like to believe the result with a high prediction probability provided by the model and hence enhance their diagnostic confidence, and if the heatmap shows the gallbladder area was highly concerned, radiologists would also prefer to trust the result provided by the model. The combination of heatmap and prediction probability could enhance the transparency and interpretability of the model.

Expert-level deep-learning systems can aid doctors by offering second opinions [29], which is confirmed in this study. Our results showed that for radiologists with limited experiences, the juniors seem to benefit more from the model than the seniors. The possible reason is that senior radiologists prefer to believe their own diagnosis due to their expertise. On the contrary, junior radiologists are more likely to trust the smartphone app model because of its expert-level performance. Nevertheless, the diagnostic confidence of BA in both junior and senior radiologists increased greatly with the aid of the smartphone app model. In addition, the diagnostic confidence of experienced pediatric radiologists had also been enhanced with the assistance of the model. This is very important because it might reduce the missed diagnosis of BA and avoid unnecessary further invasive procedures (i.e., liver biopsy) in infants with suspected BA.

In this study, we found that when the lumen of the gallbladder was small or not filled, the adjacent structures could be mistakenly regarded as gallbladder by some radiologists, especially junior radiologists. For the smartphone photos of pseudo-gallbladders, the model preferred a diagnosis of BA because these pseudo-gallbladder structures somewhat resembled abnormal gallbladders. Therefore, the application of this smartphone app will be limited in infants with a non-filled gallbladder due to the fact that most of the patients with empty gallbladder are without BA [6]. For these infants, further investigation is needed. Furthermore, image capture and interpretation might fall to different staff members in some countries. We thought that the images’ quality control should be performed by the radiologist responsible for interpreting the images, regardless of whether that radiologist is required to capture the images. This model can be applied to aid diagnosis only if the image is considered acceptable by the radiologist who interpreted it.

In clinical practice, the potential value of this model is to either improve the diagnostic accuracy or reduce the resource requirements depending on the working patterns of radiologists. If the same individual is involved in image capture and interpretation, this model may help the individual radiologist improve the diagnostic performance by providing expert-level suggestions. On the other hand, if different individuals collect and interpret data independently, this model may be expected to reduce or replace individuals who play the role of image interpretation through partially or fully automatic diagnosis. Future clinical trials can be conducted to further test the clinical application value of this model.

This study has several limitations. Firstly, nine junior and five senior radiologists participating in this study were from the same clinical center, which may introduce biases like gallbladder abnormality interpretation. The robustness of the smartphone app model in assisting radiologists from other centers needs to be tested in the future. Secondly, the diagnoses of radiologists were made by reviewing US gallbladder videos only, so the performance of radiologists could be underestimated. Additional information, such as other US features (i.e., triangular cord sign) and laboratory test results, may potentially increase the diagnostic performances of radiologists. Thirdly, no additional experiments were conducted to confirm the potential impact of the heatmap on radiologists. Further analysis is necessary to explore the relationship between heatmap attention to gallbladder features and prediction confidence in future work. Fourthly, radiologists might be dissuaded from their correct diagnoses of BA with the application of this model. Last but not least, the test cohort was collected from patients at high risk of suffering from biliary atresia but was not population-based. The proportion of biliary atresia in a real-world population-based setting would be much lower than the proportion in this study. However, it is reported that a targeted implementation in high-risk groups might be a more beneficial approach [30].

## Conclusions

The new smartphone app model showed robust and satisfactory performance for the diagnosis of BA even in the changeable environment among different operators. Most importantly, this new app could aid radiologists with limited experiences to improve their diagnostic performances and confidence in the identification of BA. It could potentially reduce the delayed diagnosis of BA for suspected infants in primary hospitals where experienced radiologists are lacking, representing considerable potential for real-world applications.

### Supplementary Information

**Additional file 1:** **Method S1. **Dataset Introduction. **Method S2. **Requirements for taking photos of smartphones. **Method S3. **Data pre-processing. **Method S4. **Model training. **Method S5. **Hospitals that provide videos to participate in clinical test. **Method S6. **Sample size calculation.** Method S7. **Sonographic gallbladder video acquisition requirements. **Table S1. **Device parameters used by 7 radiologists to take ultrasound gallbladder images with smartphones. **Table S2.** Device parameters used by 18 radiologists to take ultrasound gallbladder images with smartphones from 333 sonographic gallbladder videos. **Table S3. **Changes in the self-confidence of radiologists with different experiences in diagnosing biliary atresia with smartphone app’s assistance.** Fig. S1. **Different types of gallbladders on ultrasound. **Fig. S2. **The Confusion matrix results of the previous published model (a) and new model (b) on all external smartphone photo test data. **Fig. S3. **Example of a case where the new smartphone app helped confirm the radiologist’s diagnosis away from biliary atresia.

**Additional file 2:** **Video S1. **Normal gallbladder of a 55-day-old male infant without biliary atresia. Eighteen radiologists all correctly diagnosed it as non-biliary atresia before and after model assistance.

**Additional file 3:** **Video S2. **Abnormal gallbladder of a 46-day-old male infant with biliary atresia. Seven radiologists correctly diagnosed it as biliary atresia before and after model assistance; Six junior radiologist misdiagnosed it as non-biliary atresia initially, but finally revised it to biliary atresia with the assistance of the model; However, five radiologists misdiagnosed it as non-biliary atresia before and after model assistance.

**Additional file 4:** **Video S3. **Abnormal gallbladder of a 59-day-old female infant with biliary atresia. Eighteen radiologists all correctly diagnosed it as biliary atresia before and after model assistance.

**Additional file 5:** **Video S4. **Normal gallbladder of a 58-day-old male infant without biliary atresia. Four radiologists correctly diagnosed it as non-biliary atresia before and after model assistance; One junior radiologist misdiagnosed it as biliary atresia initially, but finally revised it to non-biliary atresia with the assistance of the model; However, thirteen radiologists misdiagnosed it as biliary atresia before and after model assistance.

**Additional file 6.** STARD 2015 Checklist.

## Data Availability

Clinical and ultrasound images are not publicly available to protect patient privacy. Original images may be made available upon reasonable request to the corresponding author (L.Y.Z.). The code is available at https://github.com/Xiangmoshihuang/BA_Deep-Learning-based-Smartphone-App.

## Abbreviations

AI: Artificial intelligence
AUC: Area under receiver operating characteristic curve
BA: Biliary atresia
CAM: Class activation map
CI: Confidence interval
US: Ultrasound
