# Supplementary material for: Interpretable artificial intelligence-based app assists inexperienced radiologists in diagnosing biliary atresia from sonographic gallbladder images
Source: BMC Med. 2024 Jan 25;22:29. doi: 10.1186/s12916-024-03247-9 (PMC10809457; doi:10.1186/s12916-024-03247-9)
Supplement: Supplementary file 1 — Additional file 1: Method S1. Dataset Introduction. Method S2. Requirements for taking photos of smartphones. Method S3. Data pre-processing. Method S4. Model training. Method S5. Hospitals that provide videos to participate in clinical test. Method S6. Sample size calculation. Method S7. Sonographic gallbladder video acquisition requirements. Table S1. Device parameters used by 7 radiologists to take ultrasound gallbladder images with smartphones. Table S2. Device parameters used by 18 radiologists to take ultrasound gallbladder images with smartphones from 333 sonographic gallbladder videos. Table S3. Changes in the self-confidence of radiologists with different experiences in diagnosing biliary atresia with smartphone app’s assistance. Fig. S1. Different types of gallbladders on ultrasound. Fig. S2. The Confusion matrix results of the previous published model (a) and new model (b) on all external smartphone photo test data. Fig. S3. Example of a case where the new smartphone app helped confirm the radiologist’s diagnosis away from biliary atresia. [file 12916_2024_3247_MOESM1_ESM.docx]

**Additional file 1**

**Supplementary Material**

**Method S1. Dataset Introduction**

In this study, both original sonographic gallbladder images and their smartphone-derived photos were employed for the new model training. The training dataset consisted of a total of 3659 original sonographic gallbladder images. Among them, there are 912 biliary atresia-related images and 2747 non-biliary atresia images. The ratio of biliary atresia to non-biliary atresia is approximate 1:3. The model was subsequently tested on 11410 external validation smartphone photos, 3374 of which were of biliary atresia and 8036 of non-biliary atresia. Ten different ultrasound devices (Mindray, Supersonic, TOSHIBA, Siemens, Samsung, HITACHI, ALOKA, Philips, GE, and Esaote) were used to capture the original US images. Both the original sonographic images and smartphone photos were stored in the 'JPEG image' format.

**Method S2. Requirements for taking photos of smartphones**

The requirements for taking photos with smartphone were as follows: (1) Making camera viewing direction perpendicular to the machine screen; (2) Including the entire gallbladder when photographed; (3) Taking photos which contain as less non-gallbladder structures as possible; (4) Focusing on the gallbladder clearly when photographed; (5) Capturing the smartphone photos in original camera mode with no filters; (6) (5) Using the lighting source or not according to the radiologist's habit during examination. The smartphone photos after taking were reviewed by a junior researcher (with 3 years of experience with pediatric abdominal ultrasound), and those in unqualified quality were required to be re-photographed. Photo images in which the gallbladder could not be identified by human eyes were considered unqualified. Otherwise, they were regarded as qualified.

**Method S3. Data pre-processing**

Due to the manual collection process of smartphone photos, additional noise would be inevitably introduced, leading to a reduction in the quality of images. Therefore, data pre-processing of these photos was required before training the model. There were two main steps involved: (1) Cropping - extracting one Region of Interest (ROI) from the smartphone photo; (2) Data augmentation - performing data augmentation on the cropped image.

In the first step, each smartphone photo was first transformed to the gray-scale image, and the single-channel gray image was expanded into three-channel gray image. Subsequently, the well-trained segmentation model (DeepLabv3 architecture) was employed to automatically crop one region of interest (ROI), i.e., the gallbladder region. The segmentation model achieved well segmentation outcomes, demonstrating a Mean Intersection over Union (Mean IoU) of 0.797 and a Mean Pixel Accuracy (mPA) of 0.921 on the gallbladder segmentation validation set. This step was to enhance the model's focus on the gallbladder region, thereby improving classification performance.

In the second step, several data augmentation techniques were implemented on the training dataset, including RandomResizedCrop [size=(224, 224), scale=(0.8, 1)], RandomHorizontalFlip (p=0.5), and ColorJitter (with a contrast factor of 0.5). These methods were utilized to augment the training dataset, consequently enhancing the model's generalization capabilities.

**Method S4. Model training**

In this study, a deep convolutional neural network (Se-ResNet-152) [22] was adopted as the architecture of the intelligent diagnosis model of biliary atresia (BA). SE-ResNet-152 is a deep learning model that integrates the strength of the ResNet architecture with the innovative Squeeze-and-Excitation (SE) block. The adopted SE-ResNet has a total depth of 152 layers, including 50 ResNet residual block and SE blocks incorporate within each residual block. The SE block comprises two key operations: the Squeeze Operation and the Excitation Operation. In the Squeeze Operation, global average pooling compresses the spatial dimensions of each feature map into a channel descriptor. Following this, the Excitation Operation models inter-channel dependencies through two fully connected layers with ReLU activations. The result is a set of channel-wise scaling factors, applied element-wise to the original feature maps. Developed to enhance feature representation and channel-wise adaptability, this model stood out for its exceptional performance in various computer vision tasks, especially biliary atresia diagnosis task. In our work, the Se-ResNet model was pre-trained on the ImageNet dataset to complete the initialization of new model parameters. In addition, Se-ResNet152, EfficientNet-b3 and EfficientNet-b5 were selected for comparative experiments on the model architectures. Pre-experimental results showed that Se-ResNet152 achieved better performance than EfficientNet-b3 and EfficientNet-b5.

During the training of the new model, the training set was first divided into five patient-level complementary subsets. Then, four subsets were used sequentially for model training, and the remaining subset was used as the internal validation set. The best performing model on the internal validation set (the model with the highest sensitivity when the training epoch > 30 and the specificity >85%) was selected as the individual model for subsequent model ensemble. The above process was repeated five times, each time with a unique subset as the internal validation set and the other four subsets as the training set, resulting in five intelligent diagnosis models of BA. For each test image, each of the five models would output the probability prediction of the test image being BA, and thresholding the probability prediction by 0.5 would lead to the specific binary predictions for each image. Majority voting over all the five models’ binary predictions was used to predict the class label of each image in either the internal or the external validation dataset. When there exist multiple images from one patient, the final specific binary prediction for each patient can be obtained by majority voting over binary predictions of these images from the five models.

The PyTorch deep learning framework was mainly used in this study. In addition, several important third-party libraries, including torchvision, numpy, pandas and scikit-learn, were also used in the study.

**Method S5. Hospitals that provide videos to participate in clinical test**

Sonographic gallbladder videos used as real-world mimic settings test set were collected from: (1) the First Affiliated Hospital of Sun Yat­sen University; (2) Shenzhen Children's Hospital; (3) Fujian Provincial Maternity and Children’s Hospital; (4) West China Hospital, Sichuan University; (5) Dongguan Children's Hospital; (6) Union Hospital, Tongji Medical College, Huazhong University of Science and Technology; (7) Tianjin Children's Hospital.

**Method S6. Sample size calculation**

In the final training of the new model, the ratio of original to smartphone-captured images was determined to be 1:14 according to the pre-experimental findings, which showed that the model's performance could be optimally enhanced after augmenting the training set with this ratio. The weighted cross-entropy loss method was used to address the issue of sample imbalance. This method assigns higher weights to categories with limited data, allowing the model to prioritize and focus on these categories.

The primary end point of the clinical tests was to evaluate the performance of the new model in assisting radiologists in diagnosing BA. We calculated the sample size using the comparison of the area under a receiver operating characteristic curve with a null hypothesis value. For α-level we selected 0.05 and for β-level we selected 0.20. According to the pre-experiment results, the area under the receiver operating characteristic curve (AUC) of the radiologist’s diagnosis with the assistance of the new App model was 0.85, and the AUC of the radiologist’s diagnosis alone was 0.75 respectively. We selected 1 for ratio of sample sizes in non-BA/BA groups. After calculation, 114 cases were required in the BA group and 114 in the non-BA group, for a total of 228 cases. Finally, we collected a total of 333 sonographic gallbladder videos (189 videos belonged to infants with BA and 144 videos belonged to 88 infants with non-BA.), and used all of them for the test in real-world mimic settings.

**Method S7. Sonographic gallbladder video acquisition requirements**

The video acquisition requirements were as follows: (1) To include only the gallbladder and not the live hilum in the field-of-view; (2) The visualization of gallbladder was clear; (3) The largest section and the whole volume of the gallbladder was included simultaneously (only subcostal acquired); (4) The depth of the image was between 4-6 cm. Each video was stored for about 5-10s for analysis. All gallbladder videos were stored in AVI format.

**Table S1 Device parameters used by 7 radiologists to take ultrasound gallbladder images with smartphones.**

| Radiologist | The brand of the smartphone | Pixels of the rear camera (million pixels) | The brand of the computer |
| --- | --- | --- | --- |
| 1 | Huawei P10 | 12 | ASUS |
| 2 | iPhone 7plus | 12 | ASUS |
| 3 | iPhone xs | 12 | Honor |
| 4 | Redmi note8 | 16 | ASUS |
| 5 | OnePlus 5 | 16 | Acer |
| 6 | Huawei nova4 | 20 | ASUS |
| 7 | OPPO rena3 | 48 | Dell |

**Table S2 Device parameters used by 18 radiologists to take ultrasound gallbladder images with smartphones from 333 sonographic gallbladder videos.**

| Radiologist | The brand of the smartphone | Pixels of the rear camera (million pixels) | The brand of the computer |
| --- | --- | --- | --- |
| A | Huawei nova7 | 64 | Dell |
| B | iPhone 12 | 12 | Razer |
| C | iPhone 12mini | 12 | Lenovo |
| D | Redmi K30 5G | 64 | Lenovo |
| E | iPhone 13 | 12 | Huawei |
| F | Honor v30 Pro | 32 | Lenovo |
| G | iPhone 13 | 12 | Lenovo |
| H | Huawei mate 40 Pro | 50 | Lenovo |
| I | iPhone 14 | 48 | MacBook Pro |
| J | iPhone 11 | 12 | Lenovo |
| K | Huawei mate10 | 12 | Lenovo |
| L | iPhone 13 | 12 | Lenovo |
| M | iPhone XS Max | 12 | Lenovo |
| N | iPhone 11 | 12 | Lenovo |
| O | Huawei P40 | 50 | Assembly computer |
| P | Huawei Mate40 pro+ | 50 | Honor |
| Q | Xiaomi MIX4 | 108 | Lenovo |
| R | iPhone 13 pro | 12 | Microsoft Surface |

**Table S3 Changes in the self-confidence of radiologists with different experiences in diagnosing biliary atresia with smartphone app’s assistance.**

| Radiologist | | Videos from BA Cohort (n=189) | | | |  | Videos from non-BA Cohort (n=144) | | | |
| --- | --- | --- | --- | --- | --- | --- | --- | --- | --- | --- |
|  |  | 1,2→3,4* | 3→4* | Maintained | 3,4→1,2* |  | 3,4→1,2* | 2→1* | Maintained | 1,2→3,4* |
| Junior radiologist | A | 26/41 | 40/46 | 109 | 4/148 |  | 16/27 | 28/42 | 77 | 14/117 |
|  | B | 13/28 | 40/50 | 122 | 5/161 |  | 24/41 | 27/39 | 82 | 10/103 |
|  | C | 6/20 | 51/73 | 121 | 6/169 |  | 8/27 | 16/63 | 78 | 6/117 |
|  | D | 17/37 | 63/70 | 97 | 2/152 |  | 19/51 | 41/55 | 60 | 6/93 |
|  | E | 13/20 | 68/82 | 93 | 12/169 |  | 31/49 | 45/67 | 39 | 17/95 |
|  | F | 2/15 | 21/25 | 158 | 5/174 |  | 22/40 | 28/32 | 83 | 4/104 |
|  | G | 7/27 | 19/49 | 157 | 2/162 |  | 9/46 | 12/49 | 116 | 2/98 |
|  | H | 18/42 | 12/23 | 149 | 2/147 |  | 13/42 | 9/17 | 108 | 3/102 |
|  | I | 52/69 | 84/97 | 36 | 7/120 |  | 19/38 | 46/54 | 51 | 12/106 |
| Senior radiologist | J | 7/21 | 8/11 | 165 | 8/168 |  | 14/37 | 19/24 | 98 | 6/107 |
|  | K | 53/75 | 35/105 | 89 | 8/114 |  | 14/38 | 3/12 | 104 | 8/106 |
|  | L | 3/11 | 9/99 | 173 | 4/178 |  | 13/74 | 4/53 | 125 | 1/70 |
|  | M | 27/78 | 13/92 | 136 | 3/111 |  | 7/18 | 12/39 | 113 | 8/126 |
|  | N | 4/21 | 19/35 | 165 | 0/168 |  | 4/38 | 10/45 | 120 | 2/106 |
| Experienced pediatric radiologist | O | 0/21 | 4/28 | 184 | 1/168 |  | 0/12 | 8/39 | 135 | 1/132 |
|  | P | 2/27 | 1/18 | 185 | 1/162 |  | 1/12 | 0/10 | 140 | 2/132 |
|  | Q | 0/11 | 16/22 | 167 | 4/178 |  | 3/39 | 17/25 | 108 | 6/105 |
|  | R | 4/33 | 5/40 | 179 | 1/156 |  | 0/10 | 0/30 | 142 | 1/134 |

Note: * ‘1’ to ‘4’ represent ‘definitely non-BA’, ‘probably non-BA’, ‘probably BA’, and ‘definitely BA’, respectively. The denominator represents the initial number of infants with diagnostic confidence at the end of the arrow, and the numerator represents the number of infants whose diagnosis has changed (from the end of the arrow to the tip of the arrow).

The data of the “Maintained” column represents the number of infants whose diagnostic confidence has not changed.

| 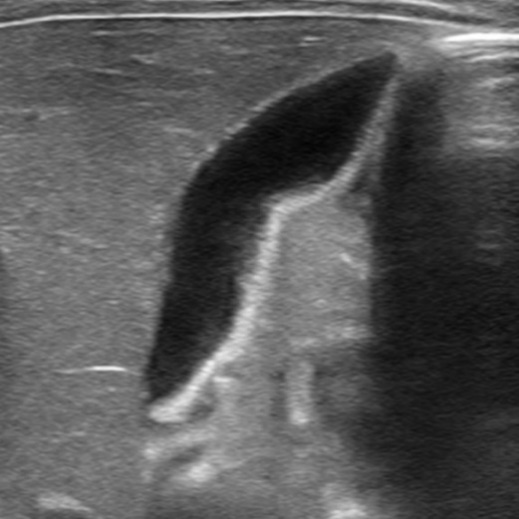  (a) | 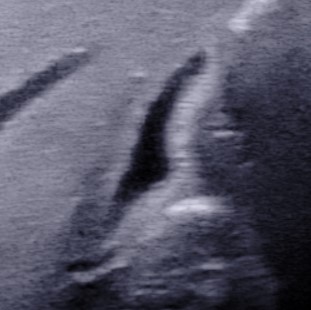  (b) |
| --- | --- |
| 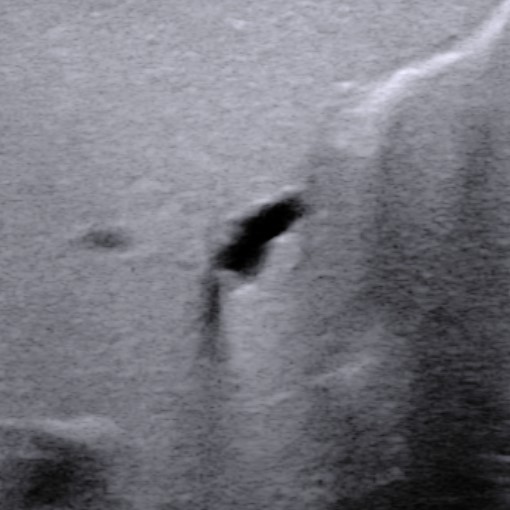  (c) | 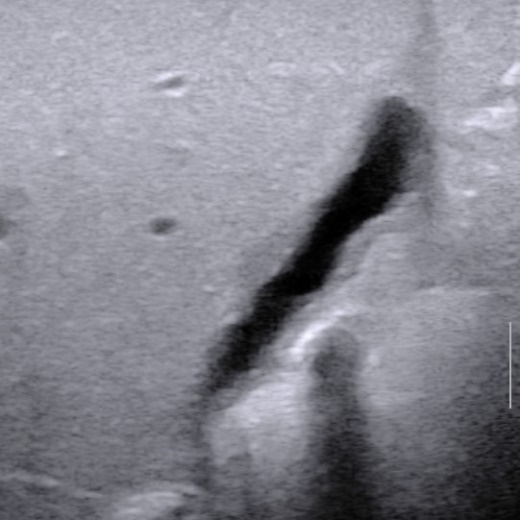  (d) |

**Fig. S1 Different types of gallbladders on ultrasound.** (**a**) a well-filled normal gallbladder with a smooth and intact echogenic mucosal lining; (**b**) a normal gallbladder with a smooth and intact echogenic mucosal lining and uniform thickening of the gallbladder wall; (**c**) an abnormal gallbladder with a fully filled lumen less than 1.5 cm in length; (**d**) an abnormal gallbladder lacks smooth echogenic mucosal lining and has indistinct wall and irregular/lobular contour.

| 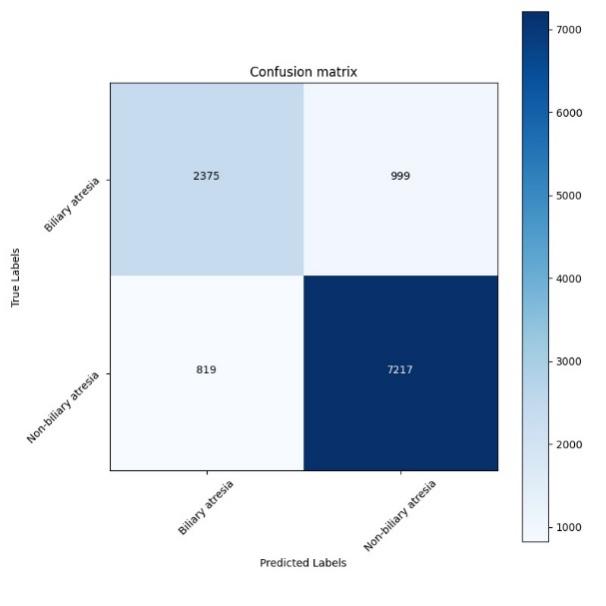  (a) | 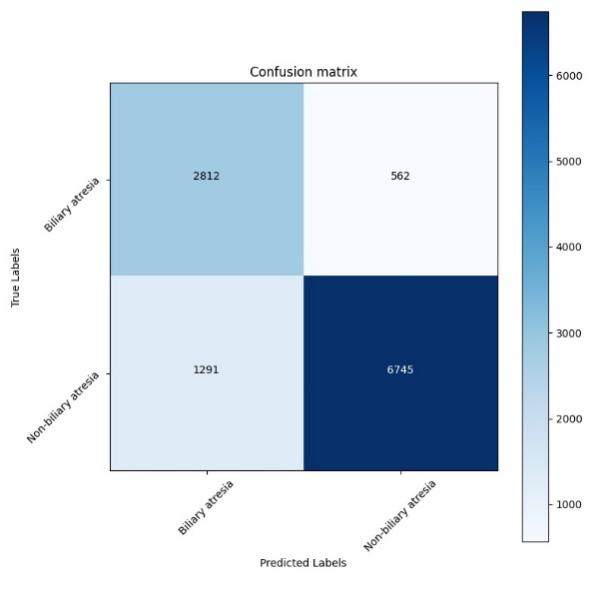  (b) |
| --- | --- |

**Fig. S2 The Confusion matrix results of the previous published model (a) and new model (b) on all external smartphone photo test data.**

| 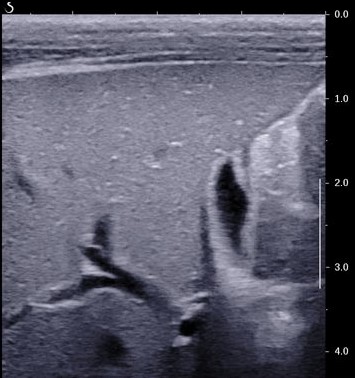  (a) | 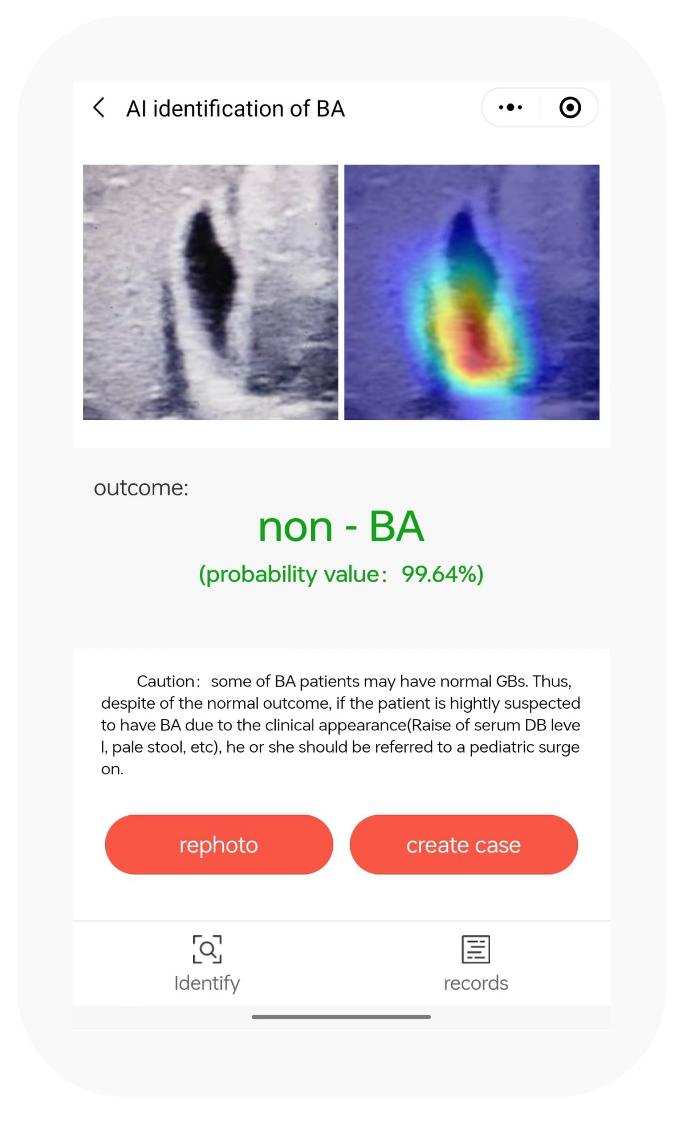  (c) |
| --- | --- |
| 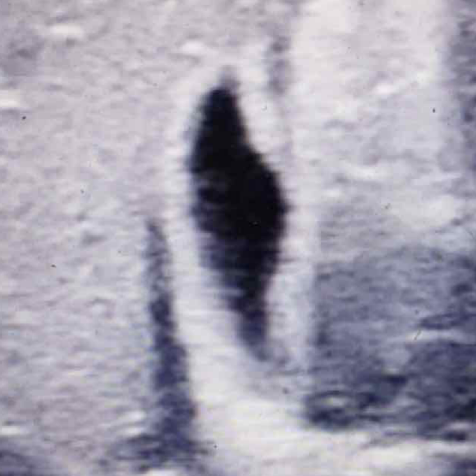  (b) |  |

**Fig. S3 Example of a case where the new smartphone app helped confirm the radiologist's diagnosis away from biliary atresia.** (**a**) An original sonographic gallbladder image selected from the video of a 78-day-old male infant without biliary atresia. 7 radiologists considered the gallbladder to be abnormal. However, the diagnosis provided by the model obtained from the photos taken by these 7 radiologists indicated non-biliary atresia, and the heatmaps all focused on the gallbladder. Finally, these 7 radiologists all revised the diagnosis of this infant to non-biliary atresia. (**b**) The smartphone photo taken by Radiologist B. (**c**) The output interface of the test result in the new smartphone app for smartphone photo shown in (**b**), displaying a probability value of 99.64%. With the assistance of the new smartphone app, Radiologists B finally revised the diagnosis of this infant to non-biliary atresia.
